# Improving the quality of barley transcriptome de novo assembling by using a hybrid approach for lines with varying spike and stem coloration

**DOI:** 10.18699/VJ21.004

**Published:** 2021-02

**Authors:** N.А. Shmakov

**Affiliations:** Institute of Cytology and Genetics of Siberian Branch of the Russian Academy of Sciences, Novosibirsk, Russia Kurchatov Genomics Center, Institute of Cytology and Genetics of Siberian Branch of the Russian Academy of Sciences, Novosibirsk, Russia

**Keywords:** RNA-seq, transcriptomics, de novo transcriptome reconstruction, IonTorrent, RNA-seq, транскриптомика, de novo реконструкция транскриптома, IonTorrent

## Abstract

De novo transcriptome assembly is an important stage of RNA-seq data computational analysis. It allows the researchers to obtain the sequences of transcripts presented in the biological sample of interest. The availability of accurate and complete transcriptome sequence of the organism of interest is, in turn, an indispensable condition for further analysis of RNA-seq data. Through years of transcriptomic research, the bioinformatics community has developed a number of assembler programs for transcriptome reconstruction from short reads of RNA-seq libraries. Different assemblers makes it possible to conduct a de novo transcriptome reconstruction and a genome-guided reconstruction. The majority of the assemblers working with RNA-seq data are based on the De Bruijn graph method of sequence reconstruction. However, specif ics of their procedures can vary drastically, as do their results. A number of authors recommend a hybrid approach to transcriptome reconstruction based on combining the results of several assemblers in order to achieve a better transcriptome assembly. The advantage of this approach has been demonstrated in a number of studies, with RNA-seq experiments conducted on the Illumina platform. In this paper, we propose a hybrid approach for creating a transcriptome assembly of the barley Hordeum vulgare isogenic line Bowman and two nearly isogenic lines contrasting in spike pigmentation, based on the results of sequencing on the IonTorrent platform. This approach implements several de novo assemblers: Trinity, Trans-ABySS and rnaSPAdes. Several assembly metrics were examined: the percentage of reference transcripts observed in the assemblies, the percentage of RNA-seq reads involved, and BUSCO scores. It was shown that, based on the summation of these metrics, transcriptome meta-assembly surpasses individual transcriptome assemblies it consists of.

## Abstract (ru)

Реконструкция транскриптома de novo – важная стадия биоинформатического анализа данных RNA- seq, которая позволяет получить последовательности транскриптов, присутствующих в изучаемом биоло- гическом образце. Наличие точной и полной последовательности транскриптома организма, в свою очередь, является необходимым условием для дальнейшей работы с данными RNA-seq. Биоинформатическим сообще- ством было создано множество программ-сборщиков для реконструкции транскриптома из коротких прочте- ний RNA-seq. Сборщики позволяют проводить как de novo реконструкцию транскриптома, так и реконструкцию, основанную на картировании коротких прочтений RNA-seq на последовательность референсного генома орга- низма. Большинство de novo сборщиков, работающих с данными RNA-seq, применяют технологию реконструк- ции последовательностей методом графов де Брёйна. Однако детали их работы могут существенно различаться, поэтому различия могут встречаться и в результатах. Некоторые авторы рекомендуют для получения более пол- ной и качественной сборки использовать гибридную сборку транскриптома – подход, основанный на комби- нации результатов работы нескольких сборщиков. Преимущество такого подхода было продемонстрировано в ряде исследований по анализу транскриптомов на платформе Illumina. Нами предложен гибридный подход по созданию сборок транскриптома ячменя Hordeum vulgare изогенной линии Bowman и двух почти изогенных линий, полученных на основе Bowman и контрастных по окраске колоса, используя данные, полученные при секвенировании матричной РНК на платформе IonTorrent. В данном подходе применяются несколько индиви- дуальных сборщиков: Trans-ABySS, rnaSPAdes и Trinity. Были оценены некоторые показатели, характеризующие полноту и точность сборки: доля обнаруженных в сборке известных транскриптов ячменя, доля задействован- ных в сборке прочтений из библиотек RNA-seq, значение критерия BUSCO. По совокупности этих показателей метасборки демонстрируют более высокое качество полученного транскриптома по сравнению с индивидуаль- ными сборщиками.

## Introduction

Next generation massively parallel sequencing technology applied to RNA (RNA-seq) is a method of choice in modern transcriptomics researches. It involves several steps: extraction of total mRNA of a biological sample, fragmentation of mRNA and simultaneous sequencing a large number of obtained short fragments (Engström et al., 2013; Hrdlickova et al., 2017).

De novo transcriptome assembly from sequenced fragments is one of the most important stages of transcriptome profiling experiment (Chang et al., 2014). It allows researcher to obtain sequences of mRNA molecules from the studied sample. Presently there are two main approaches to transcriptome sequences reconstruction from short read libraries – so-called OLC method (Overlap–Layout–Consensus) and de Bruijn graph method (Li et al., 2012; Schliesky et al., 2012). OLC method is based on pairwise alignment of reads and construction of oriented graphs where each node is one read. Overlaps between reads represent edges of the graph. This method is more suitable for contig assembly from a relatively smaller number of long reads with large overlapping regions, and thus is more frequently used to assemble sequences obtained with Saenger sequencing method or third generation sequencing methods (Cui et al., 2020).

The other method is based on construction of de Bruijn graph in which nodes are represented by k-mers – nucleotide sequences of given length k. Next, all paths on the graph that comprise sequences of short reads in RNA-seq libraries are marked. Then, all paths on the graph that contain continuous sequences of overlapping reads are marked. Thus, sequences of contigs consisting of short reads from the libraries are obtained. This method is implemented in several assemblers, namely Trinity (Grabherr et al., 2013), Trans-ABySS (Robertson et al., 2010), SOAPdenovo-Trans (Xie et al., 2014), Oases (Schulz et al., 2012).

An important parameter for de Bruijn graphs-based assemblers is k – length of k-mers used in de Bruijn graph construction. k-mers are words located in the nodes of de Bruijn graph. This parameter can be set by user prior to starting assembler program. Increasing k results in higher precision of assembly, but at the same time it makes it more computationally difficult (Fu et al., 2018). At larger k, the assembler might fail to detect a limited intersection between the reads, if its size is smaller than k. Often the following strategy is used: several preliminary assemblies are conducted at different values of k, then assemblies are combined, and redundancy reduction is performed, which results in a final de novo transcriptome assembly (Wang, Gribskov, 2017).

Since a large number of transcriptome de novo assemblers have been developed to date, researches were dedicated to answering the question of performance and precision of these programs. Reviews comparing different transcriptome assemblers usually mention Trinity, SOAPdenovo-Trans, Velvet-Oases among the best and most popular tools (Jain et al., 2013; Honaas et al., 2016; Wang, Gribskov, 2017). Trinity distributive, aside from the assembler itself, includes a large number of utilities for assembly quality assessment, removal of poorly represented contigs and other manipulations with the de novo assembly. SOAPdenovo-Trans is mentioned as the program fitting for large plant transcriptomes de novo assembly (Payá-Milans et al., 2018).

Given the diversity of modern assemblers, none of them are perfect and capable to satisfy all the requirements for completeness and quality of the assembly. Thus, it was proposed that implementing several de novo assemblers followed by creating of a single ‘meta-assembly’ may further increase sensitivity and precision of transcriptome sequences reconstruction (Cerveau, Jackson, 2016). Meta-assembly is then defined as a junction of all the de novo assemblies obtained with different tools after redundancy reduction. Redundancy reduction is a procedure of removal every contig that is a substring of at least one other contig in a given set. This approach was earlier tested for transcriptome assembly of non-model species using three assemblers – Trinity, Trans-ABySS and rnaSPAdes (Evangelistella et al., 2017). Furthermore, attempts were undertaken to obtain meta-assembly of transcriptome based on genome-guided assemblies (Venturini et al., 2018).

However, to the best of our knowledge, no attempts were made to evaluate performance of this approach on data obtained with IonTorrent sequencing platform. Meanwhile, Ion- Torrent platform, although being less popular than Illumina, is still in demand in biological researches, including studies of microbial metagenomes (Lee et al., 2019), interspecific diversity of earthworms (Shekhovtsov et al., 2019), transgenic rat lines (Bürckert et al., 2017), sequencing plant genomes (Salina et al., 2018). Furthermore, studies on comparison of Illumina and IonTorrent platforms have been performed that show IonTorrent reads having somewhat lower quality and precision that Illumina reads, and have greater discrepancy of read lengths (Lahens et al., 2017).

This research aims to create a computational pipeline based on transcriptome meta-assembly creation using de novo assemblers Trinity, Trans-ABySS and rnaSPAdes, as well as genome- guided version of Trinity based on reference genome. Computational pipeline was tested on transcriptome assembly of Hordeum vulgare L. barley isogenic line Bowman and nearly-isogenic lines iBwAlm with partial albinism of the spike and BLP with partial melanism of the spike. It was observed that quality of the transcriptome assemblies performed with different tools vary; however, in general they complement each other. Highest quality is observed for the transcriptome metaassembly, which outstrips individual assemblies based on a number of metrics that characterize overall assembly quality.

## Materials and methods

**Short read libraries.** Libraries of H. vulgare isogenic line Bowman and two nearly isogenic lines: iBwAlm (characterized by spike partial albinism) and BLP (characterized by partial melanism of the spike) transcriptome were used. The data was obtained from NCBI SRA database BioProjects PRJNA342150 (libraries of NIL i:BwAlm and isogenic line Bowman) and PRJNA399215 (libraries of NIL BLP and isogenic line Bowman).

In PRJNA342150 experiment, transcriptomes of NIL i:BwAlm, based on isogenic line Bowman, plants lemma and line Bowman, taken as a control, plants lemma were compared (Shmakov et al., 2016). For each of the lines three biological replicates were taken. Thus, in this experiment six short read libraries were sequenced. We will refer to this experiment as ‘alm experiment’ in further text.

In PRJNA399215, transcriptomes of NIL BLP, based on Bowman isogenic line, plants lemma and isogenic line Bowman, taken as a control group, plants lemma were compared (Glagoleva et al., 2017). We will refer to this experiment as ‘blp experiment’ in further text.

All libraries were obtained by sequencing using IonTorrent platform. The libraries then underwent filtration procedure, during which adapter sequences were removed using Cut- Adapt software version 1.9.1 (Martin, 2011), reads with mean quality score below 20 and lengths below 50 or above 270 were removed using Prinseq-lite software version 0.20.4 (Schmieder, Edwards, 2011). Table 1 lists metrics of the libraries used in this research.

**Table 1.**
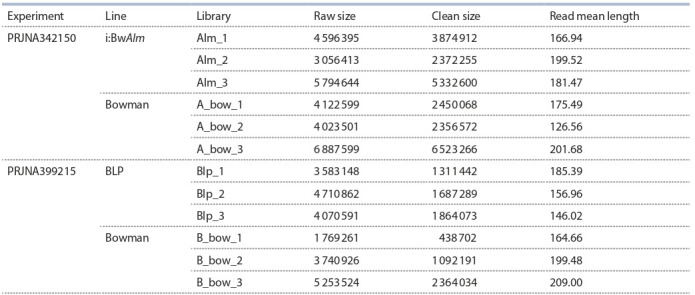
Metrics of the libraries implemented in the work

**Transcriptome reconstruction.** In this work, three transcriptome assemblers were used: Trinity (Grabherr et al., 2013) version 2.2.0, Trans-ABySS (Robertson et al., 2010) version 2.0.1 and rnaSPAdes (Bushmanova et al., 2018) version 3.12.0. All three tools were listed among the best in performance and quality in a number of researches dedicated to comparison of transcriptome de novo assemblers (Honaas et al., 2016; Lafond-Lapalme et al., 2017; Fu et al., 2018; Hölzer, Marz, 2019).

Libraries from the two experiments were processed independently. Individual transcriptome assemblies obtained with each of the software tools were reconstructed as follows.

Trinity assembler was run with default parameters; all six libraries belonging to the respective experiment were given as input files. While running SPAdes assembler, likewise, all six libraries belonging to the respective experiment were given as input files. When launching SPAdes assembler, options ‘–iontorrent’ and ‘–only-assembler’ were specified.

Trans-ABySS assembly was conducted for each of the libraries separately, with resulting assemblies combined using transabyss-merge tool from Trans-ABySS software package. This assembly was performed with default parameters, with k-mer size equal to 32. In the same way, assemblies were conducted with k-mer sizes of 48 and 64. Thus, three de novo assemblies were obtained with Trans-ABySS, differing by k- mer lengths. Next, the three assemblies were combined with transabyss-merge. Resulting assembly was further used as an individual de novo transcriptome assembly obtained with Trans-ABySS software.

Additionally, genome-guided transcriptome assembly was performed using Trinity software. First, short read libraries were mapped to barley genome. Mapping files in the SAM (sequence alignment/mapping) format were then concatenated using merge tool from samtools software package version 1.6 into a single alignment file combining mapping of all six libraries belonging to respective experiment. This file, together with the six libraries from the respective experiment, were processed with Trinity tool in genome-guided transcriptome assembly mode, with a specified parameter of maximal intron length of 500 000 nucleotides.

In order to remove redundancy of assemblies, tr2aacds.pl tool from software package Evidential Gene (Gilbert, 2019) version 20.05.2020 was implemented. Each of the individual assemblies was processed with this software. Thus, three non-redundant transcriptome de novo assemblies and one non-redundant genome-guided transcriptome assembly were obtained. We will further refer to the de novo assemblies as short versions of respective software names: abyss, spades and trinity assemblies constructed using Trans-ABySS, rnaSPAdes and Trinity, respectively. We will further refer to genome-guided transcriptome assembly as GG (short of genome-guided).

**Fig. 1.**
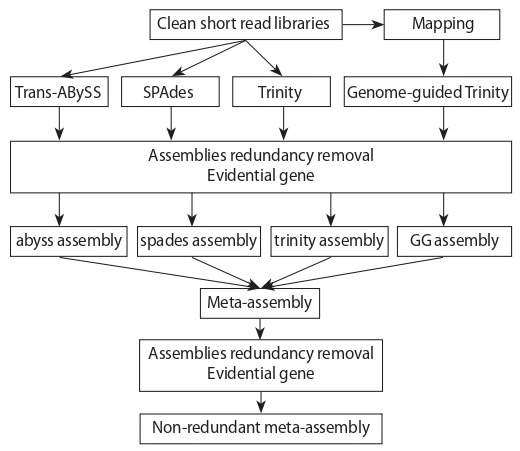
Pipeline of individual de novo barley transcriptome assemblies and barley transcriptome meta-assembly acquisition.

In order to create an optimal meta-assembly of the transcriptome, individual assemblies were concatenated into one file, which was then processed with tr2aacds.pl tool for redundancy removal. It should be noted that only contigs containing open reading frames are considered, as tr2aacds.pl only uses contigs with predicted open reading frames with length above threshold value for further analysis. Figure 1 illustrates main stages of non-redundant meta-assembly construction.

Thus, for each of the two experiments, four individual assemblies were created: spades and trinity assemblies, consisting of six short libraries belonging to the respective experiment; abyss assembly performed for each of the libraries separately with three different k-mer length values, which were later combined into a single abyss transcriptome assembly using transabyss-merge script; genome-guided GG transcriptome assembly performed on six libraries belonging to the respective experiment and alignment file combined from six libraries alignments to the barley genome. Finally, four individual assemblies for each of the experiments were combined into the barley transcriptome meta-assembly.

**Transcriptome assemblies quality assessment.** In order to analyze qualities of assemblies, each one was processed with the following tools: BUSCO (Simão et al., 2015) version 3.0.2 for completeness assessment based on presence of characteristic sequences for plants; TransRate (Smith-Unna et al., 2016) version 1.0.3 for contigs annotation and completeness of known barley genes presence in the assembly. Then, comparison of CDS lists detected by TransRate in each individual assembly was performed. Based on overlapping of the lists of CDS detected in each assembly, Venn diagrams illustrating the part of each individual assembly in the structure of meta-assembly were drawn.

Next, contigs of two meta-assemblies of barley transcriptome belonging to two experiments were aligned to the H. vulgare genome using rnaQUAST software (Bushmanova et al., 2016). rnaQUAST counts several characteristics of assembly mapping to genome, and allows the user to evaluate the assembly’s quality based on these characteristics. Specifically, this tool divides the contigs into three groups: contigs mapped to the reference and interlocking with known annotated genes; contigs mapped to the genome but lacking significant overlaps with the known annotated genes; and contigs with no homology to the known genome. We will further refer to this last group of contigs as ‘new contigs’.

**Transcriptome assemblies’ quality comparison.** In order to compare the assemblies’ quality numerically, an approach suggested in the Hölzner and Marz publication (Hölzner, Marz, 2019) was implemented. This method is to normalize a selected number of parameters that reflect de novo transcriptome assembly quality using the following formula:

**Formula 1.**
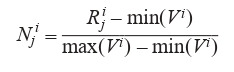
Formula 1

where R^i^_j_ is a value of parameter i for the transcriptome assembly j before normalization, N^i^_j_ is this parameter’s value after normalization, V i is a vector of all values of the parameter i for all k de novo transcriptome assemblies before normalization: V^i^ = (V^i^_1_, …, V^i^_k_). Thus, after normalization each of the parameters takes a value from 0 to 1 for each of the de novo assemblies. Next, for each of the assemblies all the normalized parameters are summed, and assemblies are sorted based on the summed values of normalized parameters. The assembly with the highest value of summed normalized parameters is considered to have the highest quality.

To compare individual assemblies and meta-assemblies of barley transcriptome obtained while working with the short read libraries belonging to two experiments, seven parameters characterizing different aspects of transcriptome assemblies were used: (1) N50; (2) median of contig lengths distribution; (3) number of BUSCO genes detected in the assembly (both complete and fragmentary genes); (4) percentage of contigs with homology to known barley CDS detected using TransRate; (5) number of barley CDS that contigs from de novo assembly are homologous with; (6) amount of barley CDS with at least 95 % of the lengths covered with aligned contigs; (7) percentage of short reads from the library that was used in construction of the de novo assembly that were mapped back to the assembly using kallisto software.

Parameters 1 and 2 reflect distribution of contig lengths. Parameters 3, 4, 5 and 6 show completeness of the transcriptome assembly. Parameter 7 shows completeness of the transcriptome assembly and how fully were the libraries used in the process of assembly construction.

## Results

**alm experiment**

For barley line i:BwAlm and control isogenic line Bowman four de novo assemblies of lemma transcriptome, and one meta-assembly consisting of the four individual assemblies were obtained. Table 2 lists results of de novo transcriptome assembly of barley lines i:BwAlm and Bowman, including common for the two lines meta-assembly.

**Table 2.**
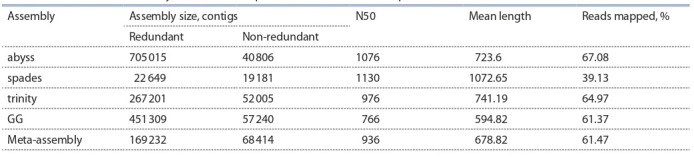
Characteristics of barley de novo transcriptome assemblies in alm experiment

Transcriptome meta-assembly of lines i:BwAlm and Bowman obtained from de novo assemblies created with rnaSPAdes, Trans-ABySS and Trinity and genome-guided Trinity assemblies, consists of 169 232 contigs before redundancy removal. Non-redundant meta-assembly consists of 68 414 contigs with total length of 46 440 750 bases. Longest contig consists of 9920 nucleotides, mean contig length is 678.8 nucleotides, N50 is 936 nucleotides. Redundancy removal reduced metaassembly size to 40.4 % of initial.

Coverage of contigs with short reads from the libraries was estimated for individual assemblies and meta-assembly of transcriptome using pseudo-alignment technique. It was observed that the highest percentage of reads was mapped to the abyss transcriptome assembly, while the lowest – to the spades assembly. 61.47 % of all the short reads were mapped to the meta-assembly of the transcriptome (see Table 2).

Search of known annotated barley CDS in transcriptome assemblies was carried out using TransRate software tool. Results of CDS identification for the assemblies are listed in the Table 3.

**Table 3.**
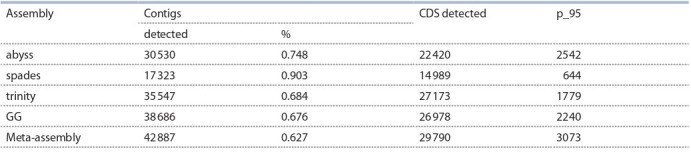
Numbers of barley CDS detected in de novo transcriptome assemblies in alm experiment

The highest amount of known CDS (29 790) was detected in meta-assembly of transcriptome. Moreover, the highest amount of CDS with coverage no less than 95 % was detected in meta-assembly. However, the highest percentage of contigs that show homology to known barley CDS was detected for the spades assembly – 90.3 %. In meta-assembly this metric is only 62.7 %, which is lower than in any of individual assemblies.

**Fig. 2.**
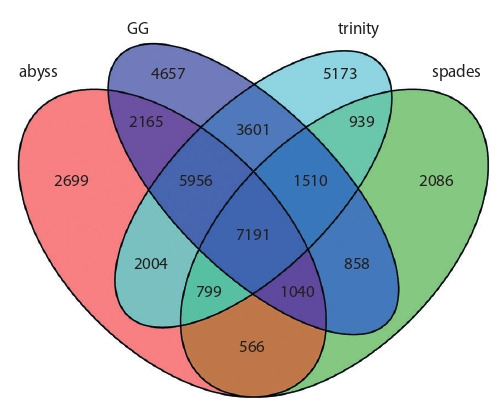
Venn diagram illustrating overlaps of CDS lists detected in individual transcriptome assemblies in alm experiment.

Furthermore, in order to estimate contribution of each of the assemblers into the transcriptome meta-assembly structure, overlapping of CDS lists detected in individual assemblies was counted. Resulting overlaps are illustrated in Figure 2. As seen from Figure 2, 7191 barley CDS were detected in all four individual assemblies; 9305 CDS were detected in three out of four assemblies. 14 615 CDS were detected in only single individual assembly, out of which the largest amount (5173) were detected only in trinity assembly, the lowest amount (2086) – only in spades assembly. The biggest intersection of CDS lists were observed between trinity assembly and GG assembly – 18 258 CDS.

**Fig. 3.**
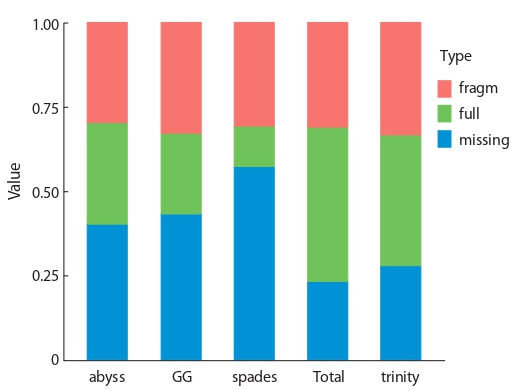
BUSCO criterion of completeness of transcriptome assembly in alm experiment.

In contigs of each of the assemblies open reading frames (ORF) were predicted. ORF detected in the contigs of metaassembly encode 58 636 protein products with lengths equal to or greater than 30 amino acid residues. These protein products were used then to evaluate integrity of the assemblies using BUSCO software, which is shown in Figure 3. Transcriptome meta-assembly contains more complete BUSCO sequences than any individual transcriptome assembly, and less fragmented and absent BUSCO sequences. This suggests that meta-assembly has higher quality and integrity.

**blp experiment**

For RNA-seq libraries from blp experiment, individual transcriptome assemblies and transcriptome meta-assembly were obtained, and quality comparison of the assemblies was performed. Table 4 lists main parameters of the assemblies.

**Table 4.**
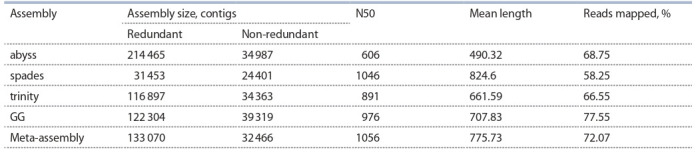
Characteristics of barley de novo transcriptome assemblies in blp experiment

Resulting transcriptome meta-assembly of barley lines Bowman and BLP consists of 133 070 contigs. After redundancy removal meta-assembly contains 32 466 contigs with total length of 25 184 753 nucleotides. Thus, redundancy removal reduced assembly size to 24.4 % of initial size. Also, it is worth noting that meta-assembly in blp experiment has a higher N50 value than any of the individual assemblies it consists of. 72.1 % of short reads from blp experiment libraries were mapped back to the transcriptome meta-assembly. For this indicator, meta-assembly is behind GG assembly (77.6 %), but ahead of three other individual assemblies.

Search of known barley CDS was carried out in transcriptome de novo assemblies of barley lines under investigation using TransRate software. Results of the search are shown in Table 5. As can be seen from Table 5, from as low as 19 848 contigs in spades assembly to as much as 29 412 contigs in GG assembly show homology to known barley CDS. Meanwhile, the highest amount of barley CDS were detected in trinity assembly, however, the highest amount of barley CDS with no less than 95 % length covered with contigs is detected in transcriptome meta-assembly – 1825 CDS. Percentage of contigs from the assembly for which homology to known CDS was detected is 74.5 % in meta-assembly which is lower that in any individual assembly except for trinity assembly.

**Table 5.**
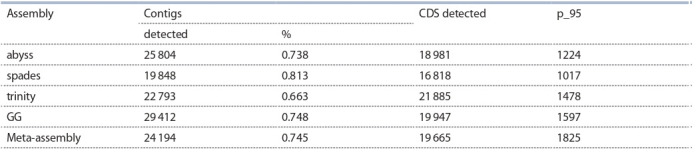
Numbers of barley CDS detected in de novo transcriptome assemblies in blp experiment

**Fig. 4.**
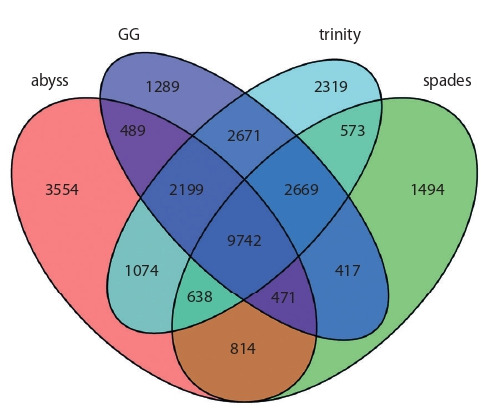
Overlaps between CDS lists detected in individual transcriptome assemblies in blp experiment.

Search of overlaps between lists of CDS detected in individual assemblies was performed, and contribution of indi- vidual assemblies into meta-assembly structure was evaluated (Fig. 4). 9742 CDS were detected in all four individual transcriptome de novo assemblies. 8656 CDS were detected in only one of individual assemblies, of which the largest amount – 3554 were unique for abyss assembly, lowest amount – 1289 were unique for GG assembly. The highest amount of common CDS is between GG and trinity assemblies – 17 281 CDS were detected in both of these assemblies.

**Fig. 5.**
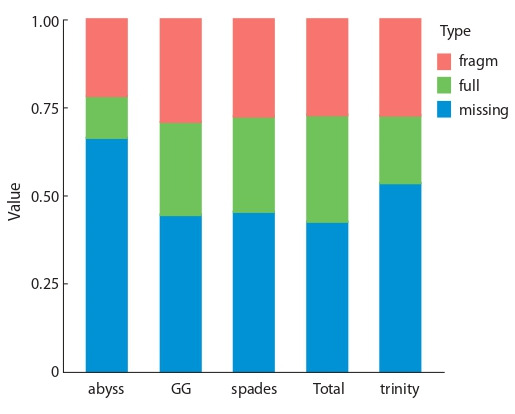
BUSCO criterion of transcriptome assembly completeness in blp experiment.

Transcriptome assemblies’ integrity estimation were carried out using BUSCO tool (Fig. 5). Meta-assembly was shown to have higher completeness than any of the individual assemblies, as it has the highest amount of complete BUSCO sequences detected and lowest amount of BUSCO sequences non-detected. In total 57.6 % of all BUSCO sequences from embryophyte set were detected in non-redundant metaassembly as completely or partially.

**Comparison of de novo assemblies’ quality**

Seven metrics of individual de novo assemblies and metaassembly were evaluated in order to assess quality of the assemblies. These metrics indicate lengths of contigs in de novo assemblies (N50 and median of lengths distribution), presence of known barley CDS in the de novo assembly (percentage of contigs with homology to known barley CDS, amount of detected CDS and amount of CDS with at least 95 % of length covered) and genes characteristic to vascular plants (BUSCO completeness criterion), and fullness of libraries short reads implementation in the assembly creation (percentage of pseudo-aligned reads). Values of these metrics were normalized and brought into the range of values from 0 to 1 (Hölzner, Marz, 2019), then sums of normalized metrics were taken for each of the individual assemblies and for the meta-assembly. The largest values of the sums show the most optimal transcriptome assembly (Table 6).

**Table 6.**
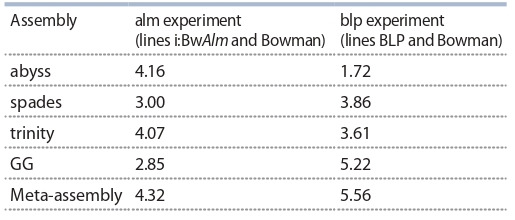
Summarized values of normalized quality metrics for de novo transcriptome assemblies in experiments alm and blp

As can be seen from the Table 6, highest values of normalized metrics are attributed to the transcriptome metaassemblies in both experiments. This, together with highest amount of detected genes characteristic to vascular plants detected with BUSCO software, and highest amounts of fully reconstructed barley CDS indicates that meta-assemblies created by combining of individual de novo transcriptome assemblies and redundancy removal outstrip individual assemblies by quality.

## Discussion

In this work, an approach to de novo transcriptome reconstruction based on creation of meta-assembly from several individual assemblies was tested. It was observed that transcriptome meta-assemblies have higher integrity judging by a number of criteria such as amount of detected BUSCO fragments, amount of barley CDS to which contigs in transcriptome assembly show homology, and percentage of pseudo-aligned to the assembly reads from RNA-seq libraries. Thus, it could be concluded that aforementioned approach to transcriptome de novo reconstruction based on creation of several individual assemblies followed by their combining into meta-assembly increases quality of de novo reconstructed transcriptome.

Comparison of several aligners showed that rnaSPAdes tool reconstructs fewer contigs, while Trans-ABySS reconstructs the highest amount of contigs. Trinity assembler reconstructs comparable quantities of contigs when run in two modes – de novo and genome-guided. At the same time, redundancy removal reduces sizes of Trans-ABySS assemblies most severely – in alm experiment 94.3 % of all contigs reconstructed by Trans-ABySS were removed, in blp experiment – 83.7 %. In the case of spades assembly, 15.3 and 22.4 % of all the contigs were removed, respectively. In trinity assemblies on average 80.5 and 70.6 % of contigs were removed, in genome-guided assemblies – 87.3 and 67.8 % of contigs, respectively. Genome-guided assemblies have the highest sizes after redundancy removal in both experiments, spades assemblies – the lowest.

Spades reconstructs the largest contigs of all individual assemblers, which is indicated by highest N50 values and medians of contig lengths distribution. Lowest N50 value in alm experiment was observed in GG assembly, in blp experiment – in abyss assembly.

The highest completeness of all individual assemblies according to BUSCO criterion in alm experiment is attributed to trinity assembly. In blp experiment it is attributed to GG assembly. The lowest completeness according to BUSCO criterion is attributed to spades assembly in alm experiment and abyss assembly in blp experiment.

## Conclusion

To conclude, in the two experiments difference in performance of the de novo transcriptome assemblers is observed, despite IonTorrent short read libraries being used in both experiments, and reconstructed transcriptome belonging to the same organism – H. vulgare barley. This suggests that implemented assemblers are sensitive to the input data, and their performance can vary depending on the data used.

However, on both accounts transcriptome meta-assemblies created from combined individual assemblies have higher quality than all individual assemblies, which indicates the effectiveness of the approach to de novo transcriptome reconstruction as building of meta-assemblies combining results of several individual de novo transcriptome assemblers.

## Conflict of interest

The authors declare no conflict of interest.
